# Supplementary material for: MetaRibo-Seq measures translation in microbiomes
Source: Nat Commun. 2020 Jun 29;11:3268. doi: 10.1038/s41467-020-17081-z (PMC7324362; doi:10.1038/s41467-020-17081-z)
Supplement: Supplementary file 10 — Supplementary Data 7 [file 41467_2020_17081_MOESM10_ESM.zip › File2/Confidence_High_Taxonomy/12418_out.krona.html]

Javascript must be enabled to view this page.

members
magnitude
magnitudeUnassigned
count
unassigned
taxon
rank

12418\_out

4

4
2
superkingdom

phylum
1239
4

4
186801
class

4
186802
order

family
541000
4

216851
4
genus


SRS024435\_contig\_number\_contig-100\_5373.40662SRS098644\_contig\_number\_contig-100\_11305.184903SRS104693\_contig\_number\_contig-100\_5800.76922SRS145308\_contig\_number\_contig-100\_13455.13456
4
853
species
